# A mouse ear skin model to study the dynamics of innate immune responses against *Staphylococcus aureus* biofilms

**DOI:** 10.1186/s12866-019-1635-z

**Published:** 2020-01-29

**Authors:** Aizat Iman Abdul Hamid, Laurence Nakusi, Mickael Givskov, Young-Tae Chang, Claire Marquès, Pascale Gueirard

**Affiliations:** 1 Laboratoire Microorganismes : Génome et Environnement, UMR CNRS 6023, Université Clermont-Auvergne, Clermont Ferrand, France; 2 Costerton Biofilm Center, department of Immunology and Microbiology, Faculty of Health Sciences, University of Copenhagen, Copenhagen, Denmark; 3 Center for Self-assembly and Complexity, IBS and Department of Chemistry, POSTECH, Pohang, Republic of Korea

**Keywords:** *Staphylococcus aureus*, Biofilm, Planktonic form, Inflammation, Mouse, Intravital imaging

## Abstract

**Background:**

*Staphylococcus aureus* is a human pathogen that is a common cause of nosocomial infections and infections on indwelling medical devices, mainly due to its ability to shift between the planktonic and the biofilm/sessile lifestyle. Biofilm infections present a serious problem in human medicine as they often lead to bacterial persistence and thus to chronic infections. The immune responses elicited by biofilms have been described as specific and ineffective. In the few experiments performed in vivo, the importance of neutrophils and macrophages as a first line of defence against biofilm infections was clearly established. However, the bilateral interactions between biofilms and myeloid cells remain poorly studied and analysis of the dynamic processes at the cellular level in tissues inoculated with biofilm bacteria is still an unexplored field. It is urgent, therefore, to develop biologically sound experimental approaches in vivo designed to extract specific immune signatures from the planktonic and biofilm forms of bacteria.

**Results:**

We propose an in vivo transgenic mouse model, used in conjunction with intravital confocal microscopy to study the dynamics of host inflammatory responses to bacteria. Culture conditions were created to prepare calibrated inocula of fluorescent planktonic and biofilm forms of bacteria. A confocal imaging acquisition and analysis protocol was then drawn up to study the recruitment of innate immune cells in the skin of LysM-EGFP transgenic mice. Using the mouse ear pinna model, we showed that inflammatory responses to *S. aureus* can be quantified over time and that the dynamics of innate immune cells after injection of either the planktonic or biofilm form can be characterized. First results showed that the ability of phagocytic cells to infiltrate the injection site and their motility is not the same in planktonic and biofilm forms of bacteria despite the cells being considerably recruited in both cases.

**Conclusion:**

We developed a mouse model of infection to compare the dynamics of the inflammatory responses to planktonic and biofilm bacteria at the tissue and cellular levels. The mouse ear pinna model is a powerful imaging system to analyse the mechanisms of biofilm tolerance to immune attacks.

## Background

*Staphylococcus aureus* (*S. aureus*) is a common commensal Gram-positive bacterium that colonizes the skin and mucous membranes of humans. It can also shift between planktonic and biofilm lifestyles and colonize abiotic surfaces such as indwelling medical devices and prosthetic implants [1]. Inside biofilms, bacteria are embedded in an extracellular matrix and are more tolerant to antibiotics and to host immune attacks [2]. The resulting impact on human health is enormous since biofilm infections account for more than 80% of microbial infections in otherwise sterile tissue(s) and often become chronic [3].

The immune responses elicited by biofilms have been described as specific and ineffective thus promoting bacterial persistence and the establishment of chronic infections [4]. Different immune evasion mechanisms have been proposed to be involved, including phagocyte direct killing (macrophages, neutrophils), specific recruitment of myeloid-derived suppressor cells (MDSCs) and macrophage polarization towards an anti-inflammatory phenotype [4, 5]. These results were mostly obtained during experiments performed in vitro in which biofilms were exposed to monocytes or neutrophils, or both [6]. In the few experiments performed in vivo with different rodent models, several parameters vary, such as the presence of a biomedical device, the tissue(s) that were inoculated or implanted with a bacteria-free or loaded device, the bacteria delivery mode and the inoculum dose [7]. These studies illustrate the importance of both neutrophils and monocytes/macrophages as a first line of defence against biofilm infections. However, the bilateral interactions between biofilms and myeloid cells remain poorly studied and analysis of the dynamic processes at the cellular level in tissues inoculated with biofilms is still an unexplored field. The mouse ear pinna is currently one of the most frequently used tissues to perform intravital confocal live imaging. In particular, it allows the analysis of cellular behaviour in an inflamed tissue [8]. We previously developed a concept that was potentially able to extract the biologically relevant features of the host and invasive bacteria after injection of either the planktonic or biofilm form of bacteria in the ear pinna [7]. In the present study, it was decided to use the transgenic fluorescent reporter laboratory mice line LysM-EGFP. Owing to the relative thinness of the ear pinna, the model enabled us to perform live imaging on recruited enhanced green fluorescent protein (EGFP) fluorescent leukocytes, in particular neutrophils and monocytes/macrophages. When the LysM-EGFP mouse ear pinna dermis was loaded with either planktonic or biofilm bacteria, the first results showed that the inflammatory response to *S. aureus* can be quantified in the skin.

Both bacterial forms induced a considerable inflammatory response at the injection site. However, real-time analysis showed different cellular dynamics with a limited access of recruited phagocytes to bacteria inside biofilms, resulting in less efficient phagocytosis. We also investigated the motility of resident or recruited phagocytes and observed that cells arrest at the injection site to interact with planktonic or biofilm bacteria. At early time points, biofilms slowed down phagocytes and modified their trajectory. Finally, the nature of the inoculum (planktonic or biofilm) influenced speed and straightness parameters differently, independently of cell-bacteria interactions at the injection site.

We therefore developed a mouse model of infection to compare the inflammatory response to planktonic and biofilm bacteria at the tissue and cellular levels. Our novel findings show that the dynamics of the inflammatory responses against the two bacterial forms are different.

## Results

### Preparation and characterization of calibrated inocula of *Staphylococcus aureus* biofilm and planktonic cultures

**Fig. 1:**
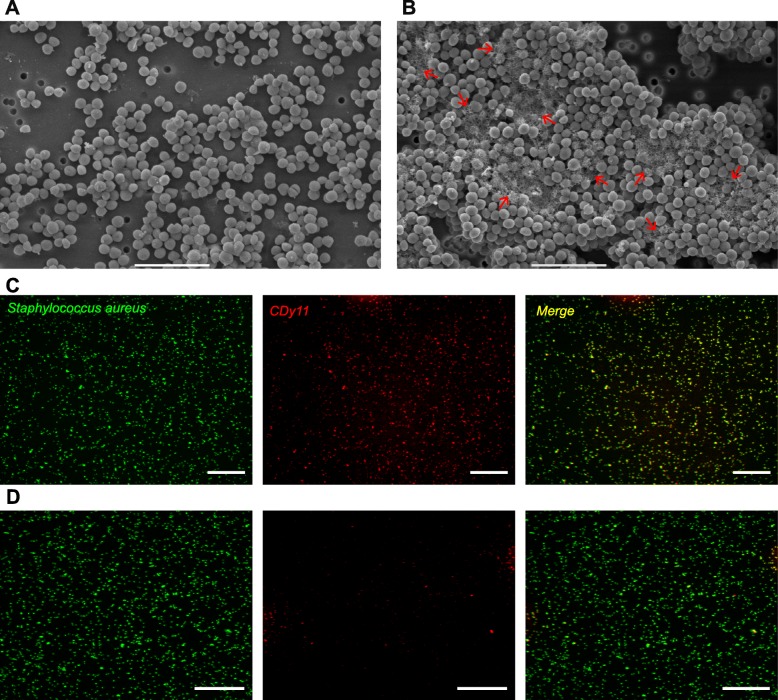
Characterization of calibrated inocula of *Staphylococcus aureus* biofilm and planktonic cultures. **a** and **b** SEM micrographs of *S. aureus* LYO-S2 planktonic (**a**) and 24 h biofilm (**b**) inocula after passing through the 34G needle used for micro-injections. Red arrows in panel B indicate the biofilm extracellular matrix. Scale bar: 5 μm. **c** and **d** Fluorescence microscopy images of *S. aureus* biofilm (**c**) and planktonic (**d**) cultures stained with the green live cell fluorescent label SYTO9 and incubated with CDy11 red fluorescent probe. Scale bar: 50 μm

A reproducible protocol of biofilm preparation was created to obtain a calibrated bacterial inoculum of 10^7^ colony-forming units (CFUs) in 3.8 μL of biofilm suspension (injection volume). As shown in Additional file 1: Figure S1A, titres of different aliquots of 24 h-old biofilms collected in the same well or in different wells for three independent experiments were comparable (Additional file 2: Table S1). To compare host immune responses to planktonic and biofilm forms of *S. aureus* LYO-S2 bacteria, calibrated inocula of planktonic bacteria were also prepared. The titres of the inocula were comparable for both bacterial forms and contained the expected quantity of bacteria (Additional file 1: Figure S1B and Additional file 2: Table S1). However, the morphological characteristics of the two inocula were different, even after passing through the 34-gauge (34G) needle used for micro-injection into the mouse ear tissue. Scanning electron microscopy (SEM) ultrastructural analysis showed that planktonic bacteria were either dispersed or organized in small clusters (Fig. 1a and Additional file 1: Figure S1C). In contrast, biofilms were organized in aggregates of 29.43 ± 7.06 μm across (Additional file 1: Figure S1D). When zoomed in, the extracellular matrix is clearly observed inside these aggregates (Fig. 1b, red arrows and Additional file 1: Figures S1E-H). However, the homogenization technique used to prepare biofilm inocula results in an inoculum containing mainly biofilm aggregates but also detached bacteria and planktonic bacteria. Future use of the term “biofilm inoculum” or “biofilm” will be in reference to this type of inoculum. Using the fluorescent probe CDy11, which targets amyloid fibrils, we observed that this biofilm matrix component was detected more abundantly in our biofilm preparations than in the samples of planktonic bacteria (Fig. 1c-d) [9].

### Micro-injection of calibrated inocula of planktonic or biofilm forms of *Staphylococcus aureus* in the mouse ear pinna induces a strong inflammatory response

**Fig. 2:**
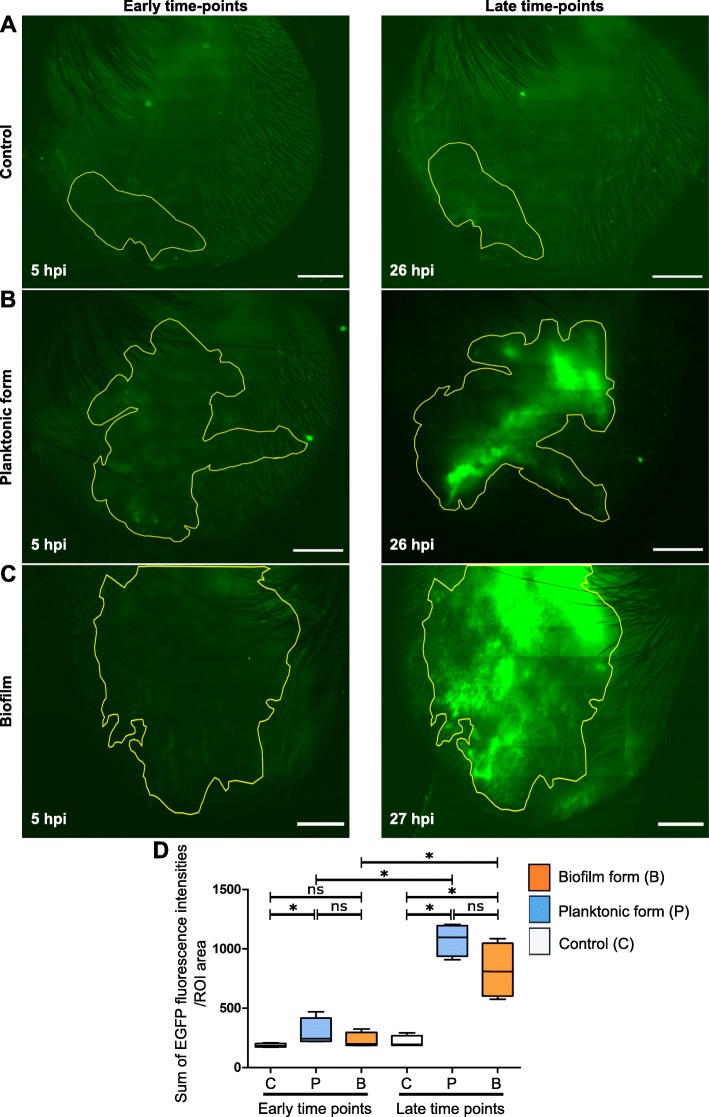
Micro-injection of calibrated inocula of *Staphylococcus aureus* in the mouse ear pinna. **a**–**c** Reconstituted confocal images of the mouse ear pinna tissue showing the maximal projection intensities of the EGFP signal. LyM-EGFP transgenic mice were micro-injected with TS culture medium (**a**) or *S. aureus* mCherry-LYO-S2 in its planktonic (**b**) or biofilm (**c**) form at early (4–7 hpi) and late time points (after 24 hpi). The EGFP fluorescence (green) signal corresponds to phagocytic cells (neutrophils and macrophages). The yellow line indicates the ROI where the “Sum of EGFP fluorescence intensities” was measured. Scale bar: 2 mm. One representative experiment is shown for each group of mice from four independent experiments. **d** Ratio of the sum of EGFP fluorescence intensities to ROI area. Data are expressed as median and interquartile ranges for four mice per group

LysM-EGFP transgenic mice were inoculated intradermally into the ear pinna with 10^7^ CFUs of either planktonic or biofilm mCherry-LYO-S2 fluorescent bacteria, or Trypticase Soja (TS) culture medium, which was used as a control. Inflammatory responses were followed at early (4–7 h post-injection [hpi]) and late time points (after 24 hpi) by measuring the intensity of the EGFP signal for each group (Fig. 2a-c). The image of the ear pinna enabled us to analyse overall inflammation in the entire tissue (mosaic acquisition). To quantify this signal, we created the following protocol. A region of interest (ROI) was drawn on late time point images, where the EGFP signal was more easily detectable, and applied to early time point images. The ratio of the sum of EGFP fluorescence intensities to ROI areas was calculated with this protocol and the inflammatory response was compared at early and late time points in the two groups of infected mice (Fig. 2d and Additional file 3: Table S2). We used the same protocol for the control group and observed a non-specific recruitment of EGFP+ innate immune cells due to the physical trauma from injection and the introduction of TS culture medium (Additional file 4: Movie S1). At early time points, both bacterial forms induced an inflammatory response, with a statistically significant increase only in the group of mice inoculated with planktonic bacteria. Thus, planktonic bacteria induced a greater response than biofilms after 4 hpi. Between the early and late time points, the inflammatory response was significantly greater for both bacterial forms. At late time points, the response was considerable in both groups of challenged mice compared to control mice, with no significant difference between mice inoculated with planktonic or biofilm bacteria (Fig. 2d).

### Dynamics of immune cell recruitment after the micro-injection of either planktonic or biofilm forms of *Staphylococcus aureus* in the mouse ear pinna are different

**Fig. 3:**
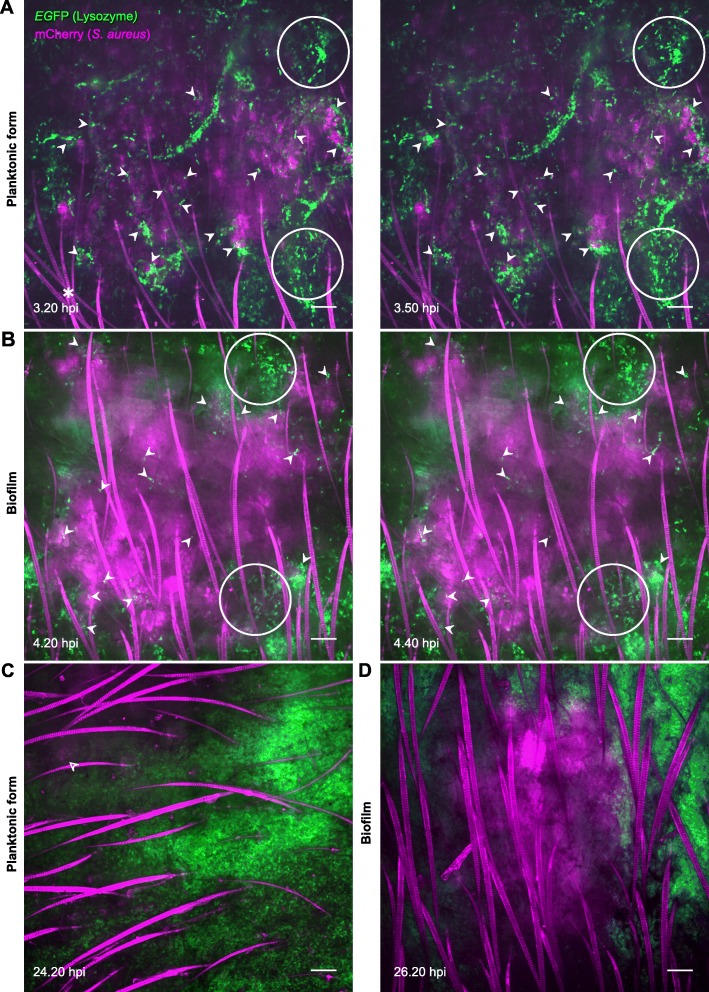
Dynamics of recruited EGFP+ cells in the mouse ear pinna after inoculation of *Staphylococcus aureus*. **a** and **b** Live confocal imaging after micro-injection of *S. aureus* mCherry-LYO-S2 in its planktonic (**a**) or biofilm (**b**) form in the ear pinna of LysM-EGFP transgenic mice at early time points. Innate immune cell recruitment towards planktonic bacteria and biofilms was observed between 3.20 to 3.50 hpi and 4.20 to 4.40 hpi, respectively. A progressive recruitment of EGFP+ innate immune cells was observed at the injection site with cell-bacteria contact areas (filled white arrowheads). White empty circles show cell accumulation over time for the planktonic or biofilm inoculum at early time points. *: autofluorescent hair (also in magenta). Scale bar: 100 μm. (**c** and **d**) Live confocal imaging at late time points after micro-injection of planktonic (**c**) or biofilm (**d**) bacteria, at 24.20 hpi and 26.20 hpi, respectively. Empty white arrowhead indicates the presence of remaining planktonic form after 24 h (low magenta signal) whereas biofilms were still easily detectable. Scale bar: 100 μm. **a–d** Images show average intensity projections of green (innate immune cells) and magenta (bacteria) fluorescence. One representative experiment is shown for each group of mice from three independent experiments

LysM-EGFP transgenic mice were inoculated intradermally into the ear pinna with 10^7^ CFUs of either planktonic or biofilm mCherry-LYO-S2 fluorescent bacteria, or with TS culture medium. We created a confocal acquisition protocol to analyse the dynamics of recruited EGFP+ cells at the inoculation sites by real-time imaging. A red autofluorescence signal is emitted by mice hairs and could not be prevented by shaving the ear pinna. Indeed, this operation would have induced a non-specific inflammatory response. In control mice, recruitment was low owing to injection trauma (Additional file 4: Movie S1). In mice inoculated with bacteria, an influx of phagocytic cells was observed at early (3 to 6 hpi) (Fig. 3a-b, white circles) and late time points (after 24 hpi) (Fig. 3c-d) for both bacterial forms. At early time points, immune cells were present over the entire surface of planktonic bacteria injection sites and multiple contact points between cells and bacteria were observed. In addition, numerous immune cells infiltrated the injection sites (Fig. 3a and Additional file 5: Figures S2A-B, white arrowheads; see also Additional file 6: Movie S2). In biofilms, the contact points were less numerous and were mainly located at the periphery of the injection site. In contrast to planktonic inocula, a small number of cells succeeded in infiltrating the biofilm (Fig. 3b and Additional file 5: Figures S2C-D, white arrowheads; see also Additional file 7: Movie S3). The fluorescent signal was less detectable for planktonic bacteria after 24 h, suggesting that bacterial lysis after phagocytosis had occurred (Fig. 3c, white empty arrowhead). For biofilms, phagocytosis seemed to be less effective, since a fluorescent signal was still clearly visible after 24 h (Fig. 3d). Overall, this real-time analysis using an intravital imaging approach shows that the dynamics of the inflammatory responses against planktonic and biofilm bacteria are different.

### Motility of recruited innate immune cells is different after injection of planktonic or biofilm forms of *Staphylococcus aureus* in the mouse ear pinna

**Fig. 4:**
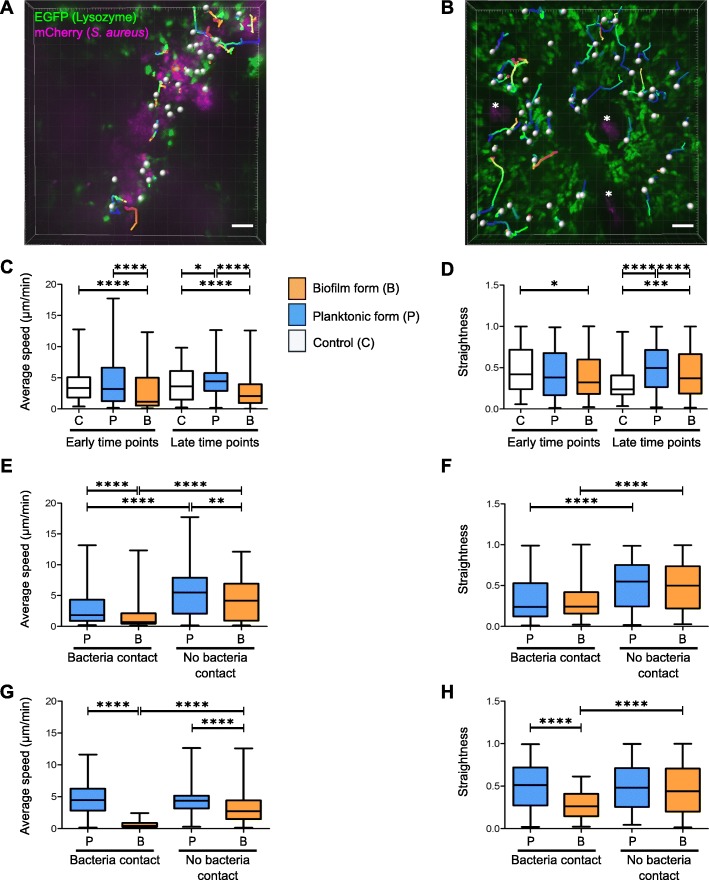
Motility of recruited EGFP+ cells in the mouse ear pinna after micro-injection of *Staphylococcus aureus*. **a** and **b** Illustration of immune cell tracking with Imaris software using the “Spots” tool to analyse the motility of recruited immune cells. The analysis was carried out in different zones of the injection site where cells were either in contact with visible bacteria (**a**) or not (**b**). Each cell is represented by a white sphere and its trajectory in the thickness of the tissue by a multicoloured line. Images shown were taken at 4.45 hpi (**a**) and 26 hpi (**b**). *: base of hair follicles. Scale bar: 50 μm. **c–h** Average speed and straightness of EGFP+ cells recruited to injection sites at early and late time points after inoculation of TS culture medium (control), planktonic bacteria (planktonic form) or biofilms (biofilm form). Data are expressed as median and interquartile ranges pooled from three different mice in three independent experiments for each group. Average speed (**c**) and straightness (**d**) of all cells (in contact with visible bacteria or not) in infected and control mice. Number of cells (N) analysed for each group at early and late time points, respectively: Control: *N* = 90 and 94 cells; Planktonic form: *N* = 315 and 433 cells; Biofilm form: *N* = 254 and 518 cells. Average speed (**e** and **g**) and straightness (**f** and **h**) of cells either in contact (bacteria contact) or not (no bacteria contact) with planktonic or biofilm bacteria at early (**e** and **f**) and late (**g** and **h**) time points. Number of cells (N) analysed at early time points that were in contact or not in contact with bacteria, respectively: Planktonic form: *N* = 157 and 158 cells; Biofilm form: *N* = 142 and 112 cells. Number of cells (N) analysed at late time points that were in contact or not in contact with bacteria, respectively: Planktonic form: *N* = 298 and 135 cells; Biofilm form: *N* = 98 and 420 cells

Using Imaris software, we created an analysis protocol to track the motility properties (average speed and straightness) of EGFP+ cells recruited at the injection zone from the previously acquired time-lapse videos (Fig. 4). Using the “Spots” tool we attributed a sphere to a number of immune cells observed in the acquisition field (Fig. 4a-b, white spheres). This enabled us to establish a trajectory (Fig. 4a-b, multicoloured lines) for each sphere corresponding to the path taken by each cell over time in the ear tissue. We then compared the average speed and straightness of the trajectories of phagocytic cells in response to the two bacterial forms. In different zones of the injection site, cells interacted with bacteria (Fig. 4a) or not (Fig. 4b). We first analysed the motility of all cells in response to bacteria (planktonic or biofilm) or to TS culture medium without distinction between cells that interacted with bacteria and cells that did not. At early time points, only biofilms induced a significant decrease in cell speed, compared to control mice and mice inoculated with planktonic bacteria. Thus, biofilms slowed down recruited cells, an effect that was maintained 24 hpi. In contrast, planktonic bacteria significantly increased cell speed compared to the control group (Fig. 4c and Additional file 8: Table S3). This differential response induced by the two bacterial forms was also seen for cell trajectory straightness, which was significantly decreased at early time points only by biofilms (less straight trajectory of EGFP+ recruited cells) compared to the control group. At late time points, we observed an opposite effect, as both bacterial forms significantly increased straightness compared to the control group, with a more pronounced effect for planktonic bacteria (Fig. 4d and Additional file 9: Table S4). We further analysed the motility of cells interacting with bacteria (bacteria contact) or not (no bacteria contact) in different zones of the cutaneous injection site for the same time point (Fig. 4e-h). Cell motility was compared after inoculation of biofilm or planktonic bacteria. At early time points, the presence of the two forms of bacteria (bacteria contact) induced a significant decrease in both speed and straightness (Fig. 4e-f, Additional file 10: Table S5 and Additional file 11: Table S6). This indicates that cells arrest at the injection site to interact with inoculated bacteria. At early and late time points, cell speed was reduced in biofilms compared to planktonic cells, independently of the presence of bacteria (Fig. 4e and g, Additional file 12: Table S7). Finally, at late time points, straightness was reduced for cells interacting with biofilms, compared to planktonic inocula (Fig. 4h and Additional file 13: Table S8). Taken together, these results demonstrate that the cell dynamics of the inflammatory response are different after inoculation of biofilm or planktonic bacteria. The mouse ear pinna model evidences an inflammatory response specific to biofilms that is probably one mechanism of its tolerance to immune attacks.

## Discussion

The dynamics of the implementation of immune responses during *S. aureus* infections in vivo is a key event. It is a determinant factor especially during planktonic-to-biofilm transition, as the bacterial persistence associated with the “chronicization” process of biofilm infection often depends on it. However, it is difficult to follow these events in mammals over time and this hinders the clear understanding of the immune evasion mechanisms of *S. aureus* biofilms and therefore the design of preventive strategies against biofilm infections.

In the present study, we compared for the first time the dynamics of early innate immune responses to planktonic and biofilm *S. aureus* in the skin. The skin is a common target tissue for *S. aureus* infections and the mouse ear pinna is frequently used as a cutaneous imaging site. This accessible tissue can be rapidly and easily prepared for imaging over long periods of time. Calibrated inocula were injected intradermally in a very small volume with a 34G needle to limit inflammation resulting from injection trauma. The maturation state of the biofilm culture and the bacterial inocula were two major criteria in the finalization of the protocol. We prepared “young” biofilms (24 h-old) as previous studies reported that immature biofilms are more susceptible to neutrophil attack than mature biofilms [10]. We inoculated a high number of bacteria (10^7^ CFUs) into the ear tissue of LysM-EGFP transgenic mice, as in previously published mouse models of *S. aureus* skin infections such as the chronic diabetic wound model and the air pouch model [11, 12]. Once drawn up, our protocol enabled us to compare qualitatively and quantitatively the innate immune responses induced by a comparable dose of planktonic or biofilm bacteria. As described above, our biofilm inoculum contained bacterial aggregates and also planktonic and detached bacteria. The phenotype of the latter is similar to that of biofilm bacteria. Indeed, previous studies have described differential gene expression profiles for *S. aureus* in its planktonic or biofilm form [13, 14]. One major difference between the two inocula was the presence of the extracellular matrix in the biofilm aggregates. Among the components of the LYO-S2 *S. aureus* biofilm matrix inoculated into mice, we detected amyloid fibrils. Small peptides called phenol-soluble modulins (PSMs) produce amyloids in *S. aureus* biofilms and notably contribute to *S. aureus* biofilm stability. They are also described as key virulence factors capable of stimulating inflammatory responses or affecting leukocyte viability or functions [15, 16], and could contribute to the specific innate immune responses observed with biofilm bacteria.

After inoculation, we used the mouse ear pinna model to follow the inflammatory response to *S. aureus* over time at the tissue level. Imaging analysis showed that bacterial inocula induced an early inflammatory response at the cutaneous injection site in LysM-EGFP transgenic mice, consisting of recruited EGFP+ phagocytes, in particular neutrophils and monocytes/macrophages. We quantified this response for the first time and showed that it is significantly increased with the planktonic form after 4 h, compared to that in control mice. The absence of significant differences between the control and biofilm conditions could have been due to early phagocyte killing, as previously reported [4, 5, 17]. After 24 h, the inflammatory response was considerable and comparable for the two bacterial forms. In rodent models, neutrophils are usually the most rapidly recruited, and therefore most abundant, cells in the proximity of biofilms [7]. In a catheter-related model, however, monocytes were the first cells to be recruited [18]. Although overall responses are comparable at late time points, we postulate that the phenotype of recruited cells in our model differs according to whether planktonic or biofilm bacteria are injected with, for example, the specific recruitment of myeloid-derived suppressor cells (MDSCs) for the biofilm, as described previously [19].

The mouse ear pinna model then enabled us to follow the inflammatory responses to planktonic or biofilm *S. aureus* over time at the cellular level. Analysis of the dynamics of recruited EGFP+ cells at the inoculation sites by real-time imaging showed that the dynamics differed between planktonic and biofilm bacteria. Our results show that biofilm acts as a physical barrier [20]. Few cells infiltrate the biofilm, with most recruited cells being present at the periphery of the inoculum. After 24 h, when innate immune responses have been considered as set up, phagocytosis seemed to be limited. Indeed, the bacterial signal was still intense, compared to the low signal observed with planktonic bacteria at the same time point. This impairment of phagocytosis observed with biofilms is commonly known as “frustrated phagocytosis” [20]. Complementary experiments are required to quantify the bacterial load in the ear tissue over time.

The mouse ear pinna model further enabled us to obtain reproducible quantitative measurements of the speed and straightness of recruited innate immune cells. To obtain the most accurate representation of these motility parameters, stringent algorithm settings were used. Then, manual corrections were applied to cell tracks. For example, only tracks lasting three or more frames were considered during the time that cells were visible in the observation field. Tracks that converged into one were eliminated to avoid any uncertainty about the resulting cell trajectory. Likewise, cells near or exiting the border of the image volume were carefully checked to ensure that the same cell was not counted twice with two different tracks. Analysis of innate immune cell migration showed that cells behaved differently in presence of planktonic and biofilm bacteria. Study of the entire population of cells in the tissue (cells interacting or not with bacteria) showed that biofilms generally decreased cell speed and straightness. In addition, when immune cells interacted with bacteria at the injection site, biofilms generally decreased cell speed more significantly than did planktonic bacteria. A possible correlation could be made with previous observations describing immobilized neutrophils on *Pseudomonas aeruginosa* biofilms in vitro after loss of their pseudopodia [21]. Interestingly, biofilms also induced a remote effect on cell speed, as cells with no visible contact with bacteria moved more slowly when the inoculum was in the biofilm form. This result suggests the potential diffusion of small molecules from the biofilm capable of influencing the behaviour of proximal recruited cells [5]. We thus provide evidence that cell motility is affected differently by planktonic and biofilm bacteria. Notably, the latter has a greater effect on speed and straightness. Further work is needed on the fine interactions between cells and bacteria in order to study phagocytic cell arrest and subsequent phagocytosis (or lack of).

## Conclusions

The mouse ear skin model proposed here detects and measures the inflammatory responses induced by biofilm and planktonic bacterial challenge over time. It has great potential to elucidate the specific mechanisms used by biofilms to circumvent host innate immune responses and therefore to develop new preventive strategies specifically targeting host immune responses during biofilm infections.

## Material and methods

### Mice and ethical statement

LysM-EGFP transgenic mice (6- to 8-week-old males and females) were obtained from the bacteria-cell interactions unit, Pasteur Institute (Paris, France), and bred in the animal care facility at Université Clermont Auvergne (Clermont-Ferrand, France). All experiments were approved by the Ethics Committee on Animal Experimentation of Auvergne C2E2A, Clermont-Ferrand, France (agreement number: 1725) and were carried out in accordance with the applicable guidelines and regulations.

### mCherry-tagged strain construction

The *S. aureus* LYO-S2 mCherry-tagged strain was constructed after insertion of pAH9 plasmid [22] into the LYO-S2 clinical strain [23] by electroporation, as described previously [24]. The *S. aureus* LYO-S2 mCherry-tagged fluorescent strain, named *S. aureus* mCherry-LYO-S2, was selected onto Luria-Bertani (LB) agar containing erythromycin (10 μg/mL). The plasmid was maintained by growing the strain in TS culture medium containing erythromycin (10 μg/mL). Fluorescence was detected in bacterial suspensions by fluorescence microscopy.

### Bacterial growth conditions

*S. aureus* LYO-S2 or the mCherry-LYO-S2 fluorescent strain were grown in TS culture medium at 37 °C with shaking and stored at − 80 °C in the same medium containing 15% glycerol. Planktonic bacteria were cultured at 37 °C in TS culture medium under aerobic conditions and harvested after overnight growth (stationary phase). For biofilm preparations, overnight cultures were adjusted to 2.10^7^ CFUs/mL of TS culture medium and added to 24-well cell culture plates (1 mL per well). Twenty-four-hour-old biofilms were obtained after incubation of plates at 37 °C without shaking.

### Preparation of bacterial inocula

Before injection, *S. aureus* mCherry-LYO-S2 planktonic inocula were prepared from the overnight growth, which was first homogenized. Bacterial concentration was then deduced by measuring the OD_600_ and using the known bacterial titre of the strain at 6.5.10^8^ CFU/OD unit. A specific volume of the overnight growth containing 10^7^ CFUs was then withdrawn and centrifuged at 3000 x g for 5 min. The supernatant was eliminated and bacteria were resuspended in TS culture medium to obtain a final concentration of 10^7^ CFUs per 3.8 μL of culture medium. For *S. aureus* mCherry-LYO-S2 biofilms, inocula were obtained by carefully eliminating 700 μL of the supernatant from each well in the cell culture plate. The remaining biofilm volume was then delicately homogenized and 3.8 μL, corresponding to 10^7^ CFUs, was collected for further inoculation to mice. Serial dilutions of both planktonic and biofilm inocula were plated on LB agar plates for titration. Biofilm inocula were sonicated three times for 5 min each before dilution (Fisher Scientific, 80 W, 37 kHz). CFUs were counted after 24 h at 37 °C.

### Inoculation of bacteria into mice

Mice were anesthetized by intraperitoneal injection of a mixture of ketamine (50 mg/kg) and xylazine (5 mg/kg). A small volume (3.8 μL) of planktonic or biofilm inocula or TS culture medium were injected into the dorsal ear dermis of anesthetized mice with a 34G needle fitted to a NanoFil syringe (World Precision Instruments) [25]. A characteristic papule was observable at the injection site, evidence of an intradermal injection.

### Scanning electron microscopy observation of bacterial preparations

For electron microscopy observations, biofilms and planktonic inocula were prepared as described above and deposited on SEM Pore (Jeol filters) with a 34G needle fitted to a NanoFil syringe. After absorption, bacteria were fixed overnight at 4 °C with glutaraldehyde 1.6% in 0.2 M cacodylate buffer at pH 7.4, supplemented with ruthenium red at 0.15%. They were then rinsed in the same buffer. After post-fixation for 1 h with 1% osmium tetroxide in cacodylate buffer at room temperature, samples were washed for 20 min in distilled water and dehydrated by graded ethanol from 25**°** to 100**°** (10 min each) to finish in hexamethyldisilazane (HMDS) evaporated overnight. After drying, samples were sputter-coated with gold-palladium (JFC-1300, JEOL, Japan). Morphology analyses were made with a scanning electron microscope JSM-6060LV (Jeol, Japan) at 5 kV in high-vacuum mode.

### Detection of amyloid fibrils in biofilm preparations

Planktonic suspensions and biofilms of *S. aureus* LYO-S2 were prepared as described previously. For planktonic bacteria, 5.6.10^8^ CFU were withdrawn from the overnight culture. The suspension was then centrifuged as before and bacteria were resuspended in 200 μL of TS culture medium. For 24 h-old biofilm cultures, 700 μL of supernatant were carefully withdrawn from the cell culture plates before homogenization of the remaining suspension. A 10 μM stock solution of the fluorescent probe CDy11 [9] was prepared in dimethyl sulfoxide (DMSO). The solution was diluted in phosphate-buffered saline solution (PBS) to prepare a 100 μM solution. Ten μL of the diluted probe was then added to each bacterial preparation and incubated for 45 min in the dark at room temperature. TS culture medium (800 μL) and 2 μL of the live cell fluorescent label SYTO9 from the LIVE/DEAD BackLight Bacterial Viability Kit (Molecular probes) were then added to each preparation and left to incubate for 15 min in the dark at room temperature. Ten μL of planktonic and biofilm preparation samples were deposited on glass slides for further observation by fluorescence microscopy. Image acquisition was carried out on a ZEISS Cell Observer Spinning Disk Confocal Microscope (Carl Zeiss Microscopy, Germany), with two different lasers to observe fluorescence emitted from SYTO9 and CDy11 (excitation at 488 and 590 nm, emission at 509 and 612, respectively, with exposure times set at 100 ms for both channels). Acquisition was performed with 20X (dry) objectives. Each image corresponds to the Z-projected average intensity signal for each channel.

### In vivo confocal imaging: acquisition

#### Time-lapse video acquisition

Three to 6 hpi, mice were anesthetized by intraperitoneal injection of a mixture of ketamine (50 mg/kg) and xylazine (5 mg/kg). Infected ears were prepared as described previously [26] and imaged on a ZEISS Cell Observer Spinning Disk Confocal Microscope (Carl Zeiss Microscopy, Germany). Video acquisition was carried out with two different lasers to observe EGFP and mCherry fluorescence (excitation at 488 and 590 nm, emission at 509 and 612, with exposure times set at 100 and 300 ms, respectively). Acquisition was performed with 10X (dry) and 20X (dry) objectives for periods of 20 to 30 min. With the 10X objective, multiple fields of observation were required as the entire injection site was imaged. Z-stacks and intervals between images were adjusted according to the thickness of the ear tissue. Acquisition was repeated 24 hpi. Ear tissues of control mice were inoculated with TS culture medium and imaged at the same time points.

#### Mosaic acquisition

Infected ears were also imaged on a ZEISS LSM 800 (Carl Zeiss Microscopy, Germany) confocal microscope with a 10X objective (dry). Multiple fields of observation covering the entirety of the tissue surface were imaged to get a reconstructed image of the ear. To set up acquisition parameters, multiple focal points distributed homogenously over the acquisition zone were chosen. EGFP fluorescence signal was detected in six Z-stacks spanning 75 μm of tissue, with an exposure time of 9.5 ms. The bright-field signal was also detected on a central stack, with an exposure time of 10 ms. Acquisition was repeated after 24 h, with imaging sessions typically lasting 30 to 45 min. Ear tissues of control mice injected with TS culture medium were also imaged with the same protocol.

### In vivo confocal imaging: analysis

#### Time-lapse video analysis

Videos acquired with the 10X objective were first stitched together using ZEN software. Each image extracted from time-lapse videos corresponds to the Z-projected average intensity signal for each channel at the corresponding time point. Time- lapse videos at 20X and 10X were then analysed with Imaris software using the “Spots” tool. For each cell, a track was generated by the software and manually corrected according to specific criteria: number of frames superior to three and elimination of converging tracks between two different cells. Two different parameters (average speed and straightness) of immune cell dynamics were then extracted. For each time point, both parameters were analysed in different zones of the cutaneous injection site, where cells were in contact or not with the bacterial inoculum.

#### Mosaic analysis

Images acquired on the ZEISS LSM 800 confocal were stitched together using ZEN software to reconstitute an entire image of the ear tissue at early and late time points. A maximum intensity projection image was created from image Z-stacks. A ROI was then drawn manually around the EGFP fluorescent zone of the 24 h image to obtain the sum of EGFP fluorescence intensities of each pixel in the ROI. The shape of the ROI was conserved and applied to the early time point image. The ratio of the sum of intensities of EGFP fluorescence to the area of the ROI was then calculated for both time points. The images shown represent the Z-projected maximal intensity signal of a reconstituted image of the ear tissue for the EGFP channel.

### Statistical analysis

Prism 5 software (GraphPad Software, Inc.) was used to analyse the statistical significance of data sets by the Mann-Whitney two-tailed test. *p* ≤ 0.05 was considered statistically significant (symbols: ****p ≤ 0.0001; ***p ≤ 0.001; **p ≤ 0.01; * ≤ 0.05; ns = non-significant).

## Supplementary information

**Additional file 1: Figure S1.** Preparation and characterization of calibrated inocula of *Staphylococcus aureus* biofilm and planktonic cultures. **(A)** Titration of 3.8 μL aliquots of 24 h-old biofilms of *S. aureus* LYO-S2. Data represent mean ± SD of three samples per well collected from three different wells and prepared in three independent experiments. **(B)** Titration of *S. aureus* LYO-S2 planktonic and 24 h biofilm inocula on agar plates. Results are expressed as CFU numbers × 10^7^ in 3.8 μL (injection volume). Data represent mean ± SD from 17 experiments for the planktonic form and from 27 experiments for biofilms. **(C)** Planktonic inocula after passing through a 34G needle. Scale bar: 10 μm. **(D to H)** Biofilm inocula after passing through a 34G needle. Red arrows indicate the biofilm extracellular matrix. Scale bar: 10 μm **(D)**, 5 μm **(E and F)**, 2 μm **(G and H)**.

**Additional file 2: Table S1.** Tables presenting raw data used for the preparation of calibrated *Staphylococcus aureus* biofilm and planktonic inocula.

**Additional file 3: Table S2.** Table presenting raw data used to measure the ratio of the sum of EGFP fluorescence intensities to ROI areas.

**Additional file 4: Movie S1.** Immune cells are recruited to injection sites even in the absence of bacterial challenge. In vivo confocal time-lapse imaging of immune cell migration in LysM-EGFP transgenic mice ear tissue injected with TS culture medium from 4 hpi to 4.20 hpi. Average projections of time-lapse images. Z-stacks collected 41.76 s apart. Scale bar: 100 μm.

**Additional file 5: Figure S2.** Dynamics of recruited EGFP+ cells in the mouse ear pinna after micro-injection of *Staphylococcus aureus*. **(A and B)** Confocal images of injection sites after micro-injection of *S. aureus* mCherry-LYO-S2 in its planktonic form in the ear pinna of LysM-EGFP transgenic mice at early time points for two independent experiments. Images of innate immune cell recruitment towards planktonic bacteria were acquired at 5.15 hpi **(A)** and 3.05 hpi **(B)**. **(C and D)** Confocal images of injection sites after micro-injection of *S. aureus* mCherry-LYO-S2 in its biofilm form in the ear pinna of LysM-EGFP transgenic mice at early time points for two independent experiments. Images of innate immune cell recruitment towards biofilms were acquired at 4.20 hpi **(C)** and 3.30 hpi **(D).** Images show average intensity projections of green (innate immune cells) and magenta (bacteria) fluorescence. Filled white arrowheads indicate cell-bacteria contact areas. *: autofluorescent hair (also in magenta). Scale bar: 100 μm.

**Additional file 6: Movie S2.** Numerous immune cells penetrate the injection site and interact with planktonic bacteria. In vivo confocal time-lapse imaging of immune cell migration in LysM-EGFP transgenic mice ear tissue injected with planktonic bacteria from 3.20 hpi to 3.50 hpi. Average projections of time-lapse images. Z-stacks collected 41.73 s apart. Scale bar: 100 μm.

**Additional file 7: Movie S3.** Most immune cells arrest at the periphery of injected biofilms. In vivo confocal time-lapse imaging of immune cell migration in LysM-EGFP transgenic mice ear tissue injected with planktonic bacteria from 4.20 hpi to 4.40 hpi. Average projections of time-lapse images. Z-stacks collected 45.15 s apart. Scale bar: 100 μm.

**Additional file 8: Table S3.** Table presenting the average speed of all cells in infected and control mice at early and late time points. Raw data extracted from Imaris software.

**Additional file 9: Table S4.** Table presenting the straightness of all cells in infected and control mice at early and late time points. Raw data extracted from Imaris software.

**Additional file 10: Table S5.** Table presenting the average speed of cells in contact with bacteria or not in infected mice at early time points. Raw data extracted from Imaris software.

**Additional file 11: Table S6.** Table presenting the straightness of cells in contact with bacteria or not in infected mice at early time points. Raw data extracted from Imaris software.

**Additional file 12: Table S7.** Table presenting the average speed of cells in contact with bacteria or not in infected mice at late time points. Raw data extracted from Imaris software.

**Additional file 13: Table S8.** Table presenting the straightness of cells in contact with bacteria or not in infected mice at late time points. Raw data extracted from Imaris software.

## Data Availability

All data generated or analysed during this study are included in this published article and its supplementary information files.

## Abbreviations

CFU: Colony Forming Unit
DMSO: Dimethyl sulfoxide
EGFP: Enhanced Green Fluorescent Protein
EGFP+: Enhanced Green Fluorescent Protein-positive
hpi: Hours post-injection
LB: Luria-Bertani
PBS: Phosphate Buffered Saline
PSM: Phenol Soluble Modulins
ROI: Region Of Interest
SEM: Scanning Electron Microscopy
TS: Trypticase Soja
