# Supplementary figures and images for: A mouse ear skin model to study the dynamics of innate immune responses against Staphylococcus aureus biofilms
Source: BMC Microbiol. 2020 Jan 29;20:22. doi: 10.1186/s12866-019-1635-z (PMC6990489; doi:10.1186/s12866-019-1635-z)

**A**

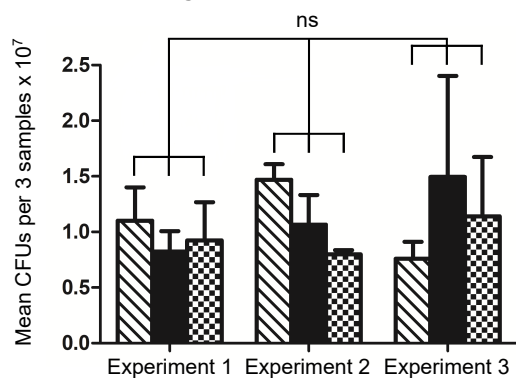

**B**

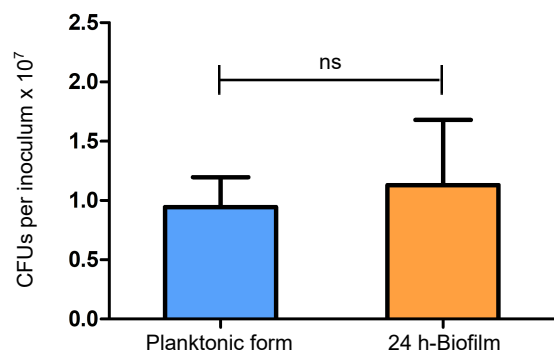

**C**

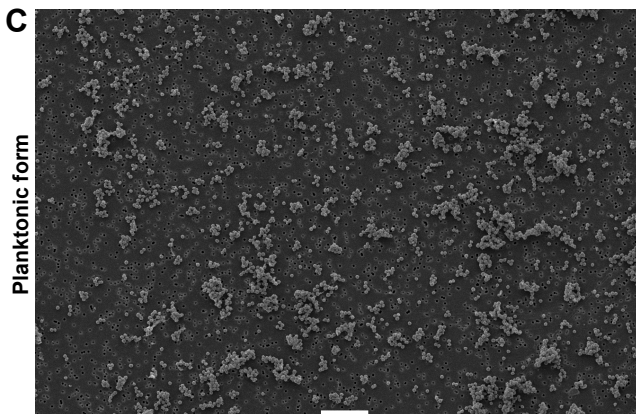

**D**

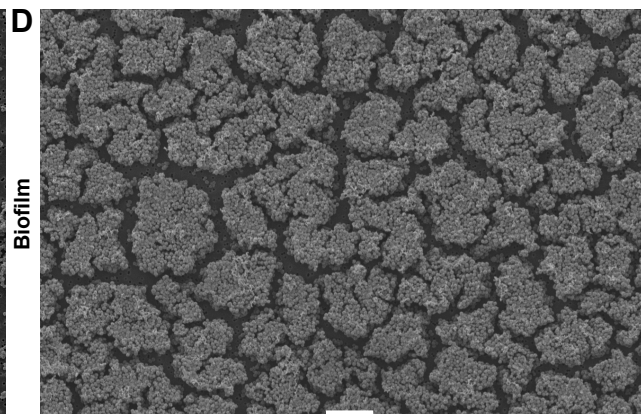

**E**

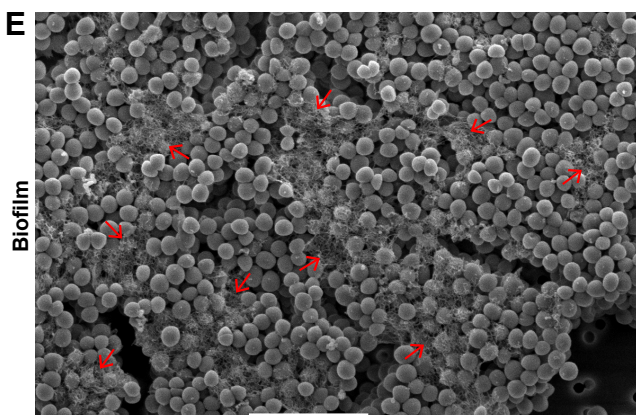

**F**

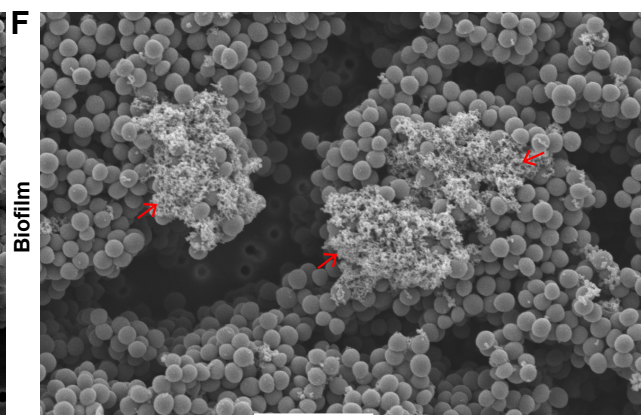

**G**

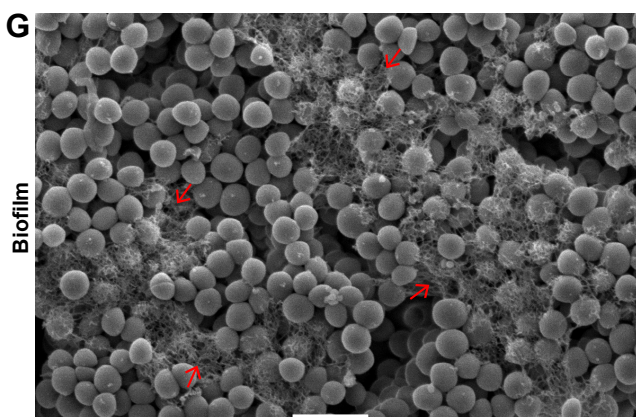

**H**

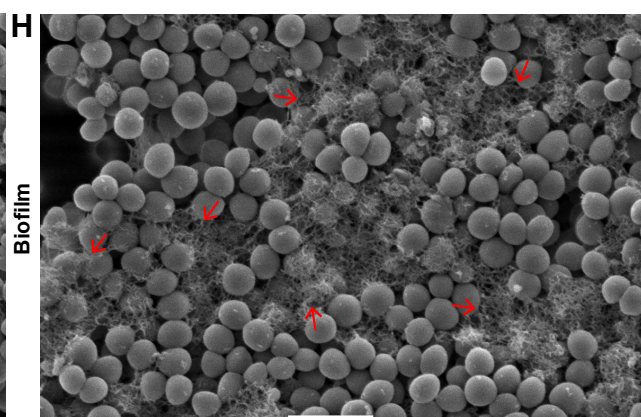

Supplement: Supplementary file 1 — Additional file 1: Figure S1. Preparation and characterization of calibrated inocula of Staphylococcus aureus biofilm and planktonic cultures. (A) Titration of 3.8 μL aliquots of 24 h-old biofilms of S. aureus LYO-S2. Data represent mean ± SD of three samples per well collected from three different wells and prepared in three independent experiments. (B) Titration of S. aureus LYO-S2 planktonic and 24 h biofilm inocula on agar plates. Results are expressed as CFU numbers × 107 in 3.8 μL (injection volume). Data represent mean ± SD from 17 experiments for the planktonic form and from 27 experiments for biofilms. (C) Planktonic inocula after passing through a 34G needle. Scale bar: 10 μm. (D to H) Biofilm inocula after passing through a 34G needle. Red arrows indicate the biofilm extracellular matrix. Scale bar: 10 μm (D), 5 μm (E and F), 2 μm (G and H). [file 12866_2019_1635_MOESM1_ESM.pdf]

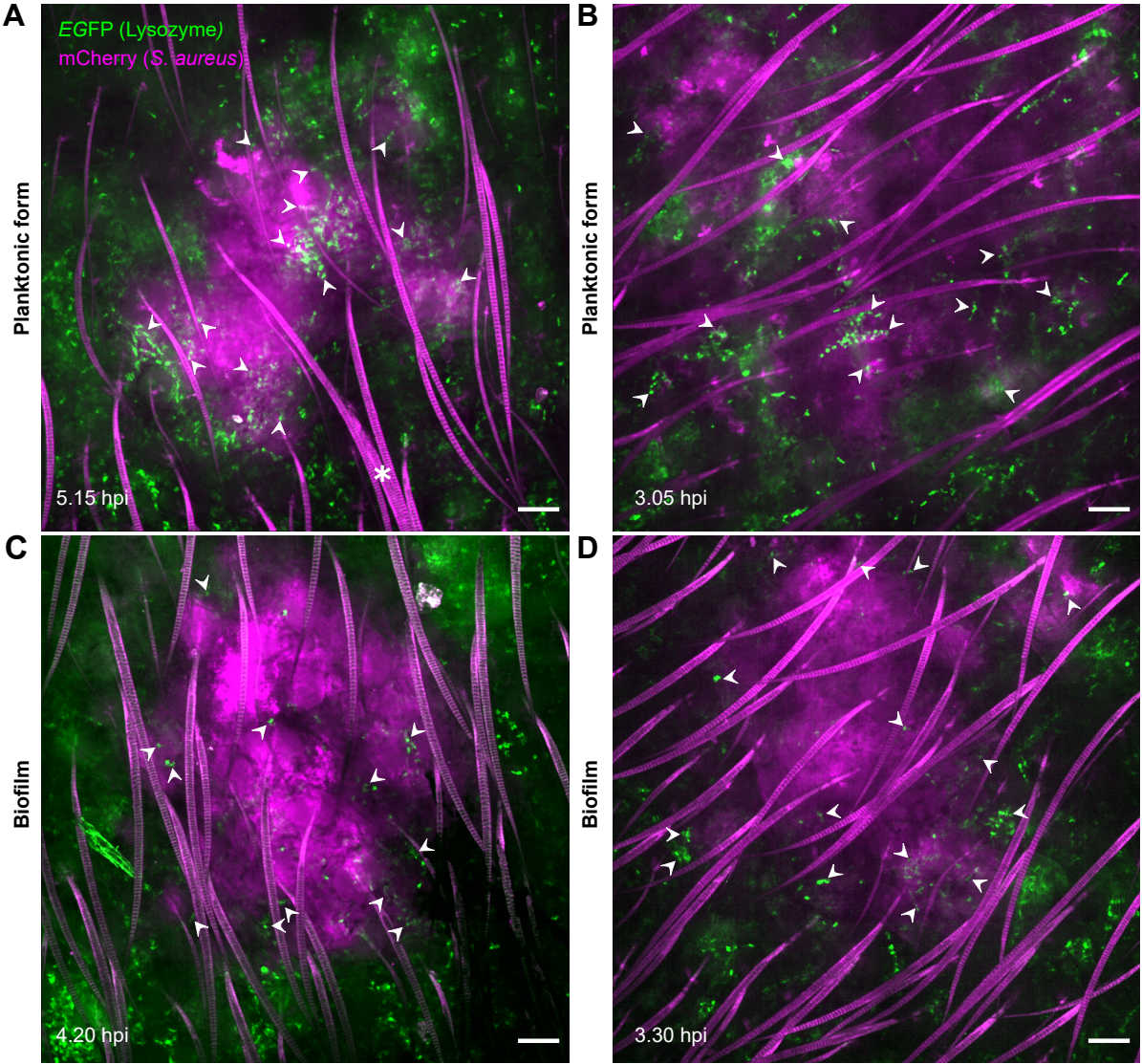

Supplement: Supplementary file 5 — Additional file 5: Figure S2. Dynamics of recruited EGFP+ cells in the mouse ear pinna after micro-injection of Staphylococcus aureus. (A and B) Confocal images of injection sites after micro-injection of S. aureus mCherry-LYO-S2 in its planktonic form in the ear pinna of LysM-EGFP transgenic mice at early time points for two independent experiments. Images of innate immune cell recruitment towards planktonic bacteria were acquired at 5.15 hpi (A) and 3.05 hpi (B). (C and D) Confocal images of injection sites after micro-injection of S. aureus mCherry-LYO-S2 in its biofilm form in the ear pinna of LysM-EGFP transgenic mice at early time points for two independent experiments. Images of innate immune cell recruitment towards biofilms were acquired at 4.20 hpi (C) and 3.30 hpi (D). Images show average intensity projections of green (innate immune cells) and magenta (bacteria) fluorescence. Filled white arrowheads indicate cell-bacteria contact areas. *: autofluorescent hair (also in magenta). Scale bar: 100 μm. [file 12866_2019_1635_MOESM5_ESM.pdf]
